# Supplementary material for: Operando dynamics of trapped carriers in perovskite solar cells observed via infrared optical activation spectroscopy
Source: Nat Commun. 2023 Dec 4;14:8000. doi: 10.1038/s41467-023-43852-5 (PMC10694143; doi:10.1038/s41467-023-43852-5)
Supplement: Supplementary file 1 — Supplementary Information [file 41467_2023_43852_MOESM1_ESM.pdf]

Supplementary Information for

Operando Dynamics of Trapped Carriers in Perovskite Solar Cells Observed via Infrared Optical Activation Spectroscopy

Jiaxin Pan[1], Ziming Chen[1,], Tiankai Zhang[2], Beier Hu[1], Haoqing Ning[1], Zhu Meng[1], Ziyu Su[1], Davide Nodari[1], Weidong Xu[1], Ganghong Min[1], Mengyun Chen[2], Xianjie Liu[3], Nicola Gasparini[1], Saif A. Haque[1], Piers R. F. Barnes[4], Feng Gao[2], Artem A. Bakulin[1]*

[1] Department of Chemistry and Centre for Processible Electronics, Imperial College London, London W12 0BZ, UK

[2] Department of Physics, Chemistry and Biology (IFM), Linköping University, Linköping SE-58183, Sweden

[3] Laboratory of Organic Electronics, ITN, Linköping University, Norrköping SE-60174, Sweden

[4] Department of Physics, Imperial College London, London SW7 2AZ, UK

* Correspondence: z.chen@imperial.ac.uk (Z.C.)

This file includes:

Supplementary Figures 1–17

Supplementary Tables 1–6

Supplementary Notes 1–6

Supplementary References

Supplementary Figures

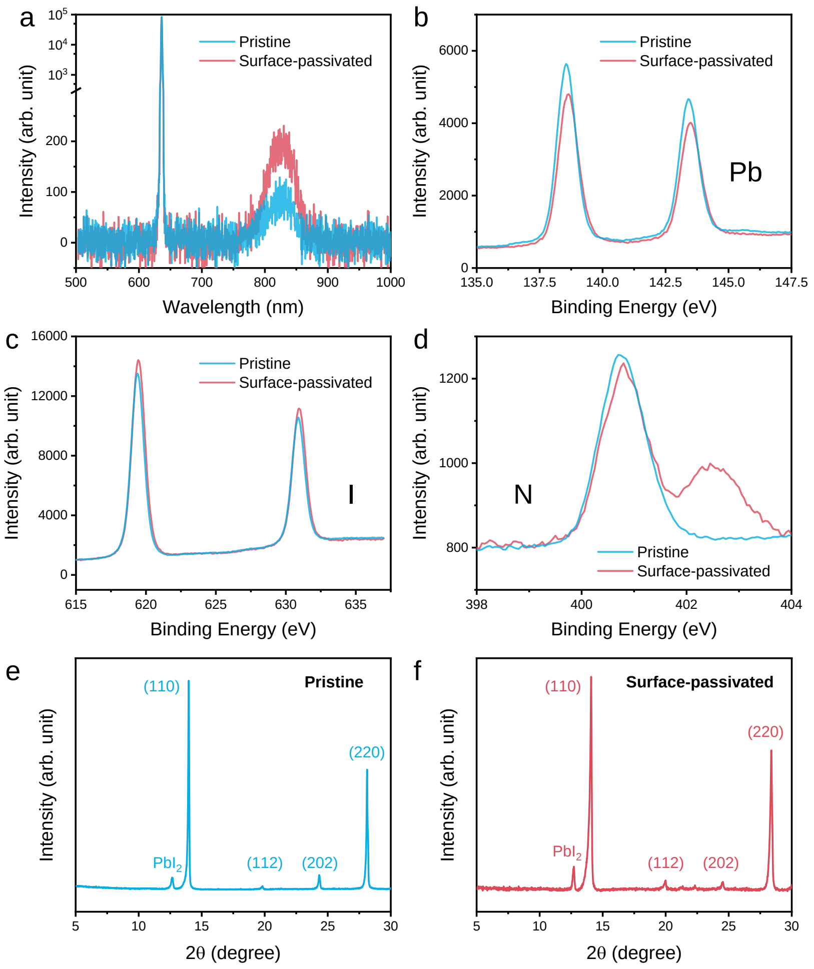

Supplementary Figure 1 | Passivation effect and impact of OAI. (a) PL quantum yield measurement of pristine (Glass/$FA_{0.99}Cs_{0.01}PbI_3$) and surface-passivated (Glass/$FA_{0.99}Cs_{0.01}PbI_3$/OAI) films. Both films were excited by a 635-nm CW laser. The PL quantum yields of pristine and surface-passivated perovskite films were 4.9% and 13.1%, respectively. XPS of (b) Pb, (c) I and (d) N in both pristine and surface-passivated films. In the pristine film, the calculated Pb:I ratio is 1:1.98, which is much larger than the ideal stoichiometry of 1:3 in perovskites, suggesting an abundance of I^- vacancies on the perovskite surface. After depositing the OAI layer, the ratio between Pb and I becomes 1:2.45. The extra I^- supplied from OAI compensates the halide vacancies. In addition, in the pristine film, the calculated Pb:N ratio is 1: 0.715, which is also larger than their ideal stoichiometry of 1:2 in $FA_{0.99}Cs_{0.01}PbI_3$, suggesting the existence of FA^+ vacancies at the perovskite surface. After depositing OAI, an extra N peak at ~402.5 eV appears (originated from the NH_3^+ in OAI) and the calculated Pb:N ratio becomes 1:1.06. This result also suggests the compensation of FA^+ vacancies by the mounting of the OA chains at the perovskite surface. Therefore, we consider OAI has a bi-functional passivation on both the FA^+ and I^- vacancies simultaneously in the studied devices. X-ray diffraction patterns of (e) pristine and (f) surface-passivated perovskite films. We consider no 2D perovskites formed as only 3D perovskite peaks are observed.

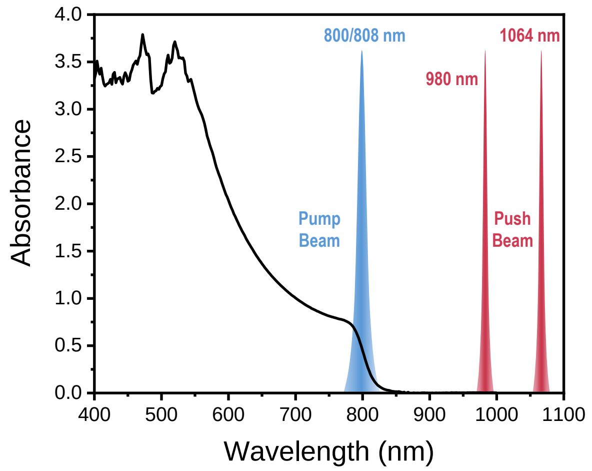

Supplementary Figure 2 | Absorption spectrum of $FA_{0.99}Cs_{0.01}PbI_3$ film and selective wavelengths of pump beam (808 nm for CW-PPPc and 800 nm for ns-PPPc) as well as IR push beam (980 nm for CW-PPPc and 1064 nm for ns-PPPc). The energy of pump and push beams is larger and smaller than the bandgap of $FA_{0.99}Cs_{0.01}PbI_3$, respectively. The energy of 980-nm (~1.27 eV) and 1064-nm (~1.17 eV) push beams is large enough to push the majority of trapped carriers located in both shallow and deep traps of perovskite.

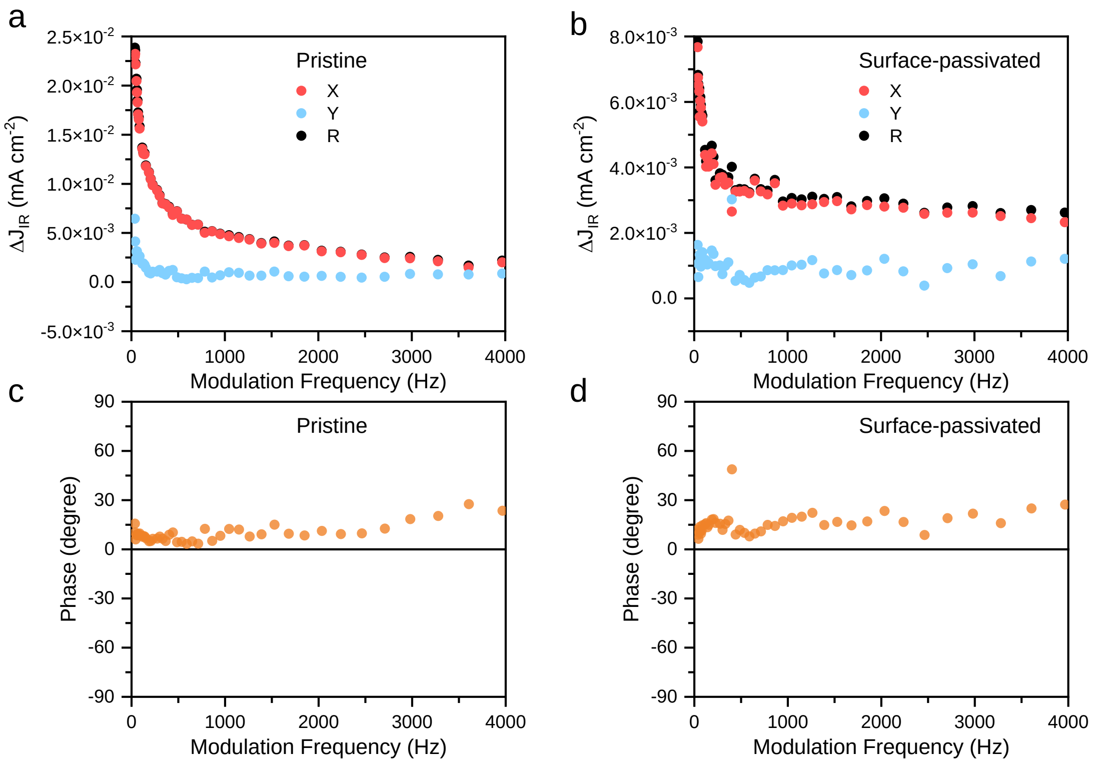

Supplementary Figure 3 | Lock-in amplifier signal as a function of modulation frequency in CW-PPPc measurement. X, Y, and R values of (a) pristine and (b) surface-passivated devices, as well as corresponding phases of (c) pristine and (d) surface-passivated devices. The phases remain approximately constant across all frequencies.

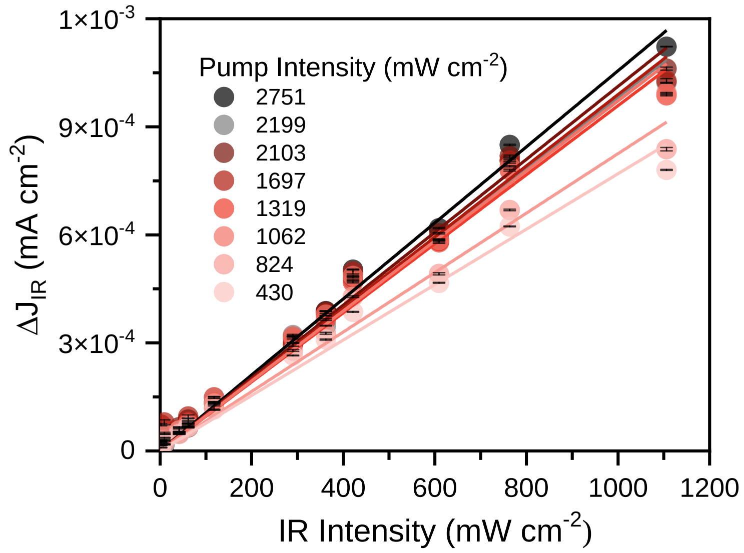

Supplementary Figure 4 | ΔJ_{IR} versus IR push intensity curves of surface-passivated devices in CW-PPPc measurement. The solid lines indicate the linearly fitted results. The error bars represent the standard deviation of the data.

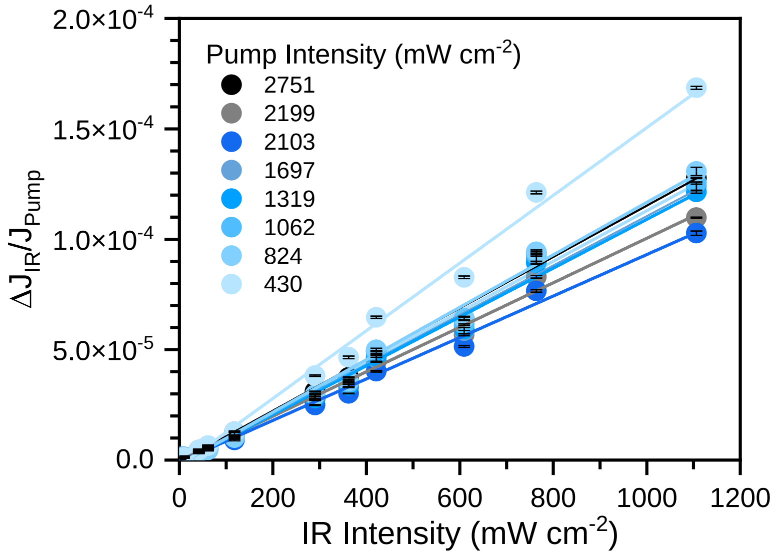

Supplementary Figure 5 | $\Delta J_{IR}/J_{Pump}$ versus IR push intensity of a pristine $FA_{0.99}Cs_{0.01}PbI_3$ device under various pump intensities. The solid lines indicate the linearly fitted results. The error bars represent the standard deviation of the data.

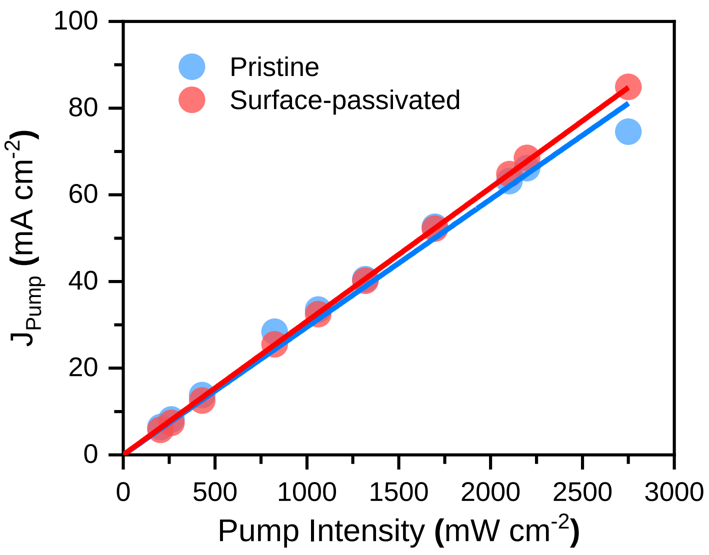

Supplementary Figure 6 | J_{Pump} versus pump intensity curves in CW-PPPc measurement of pristine and surface-passivated devices. Both curves show linear relationships, which reflects either negligible second-order recombination or pseudo-first order recombination was dominant in the devices.

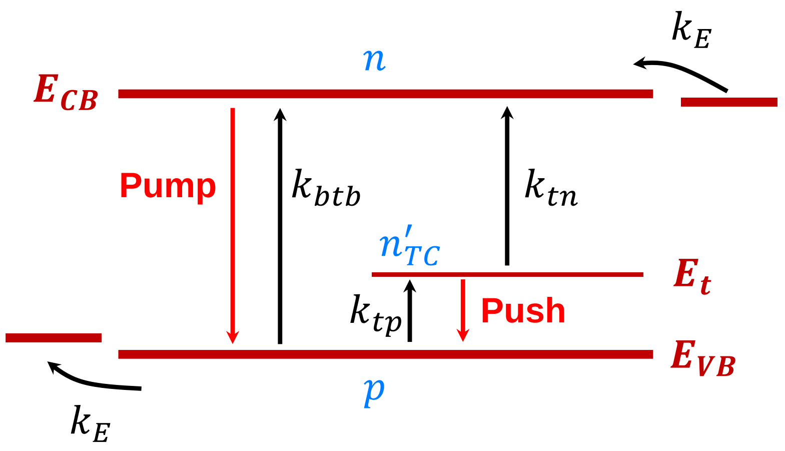

Supplementary Figure 7 | Kinetic model for the carrier trapping and recombination processes. All the arrows indicate photophysics processes in terms of holes. Detailed discussion about this model can be found in Supplementary Note 4.

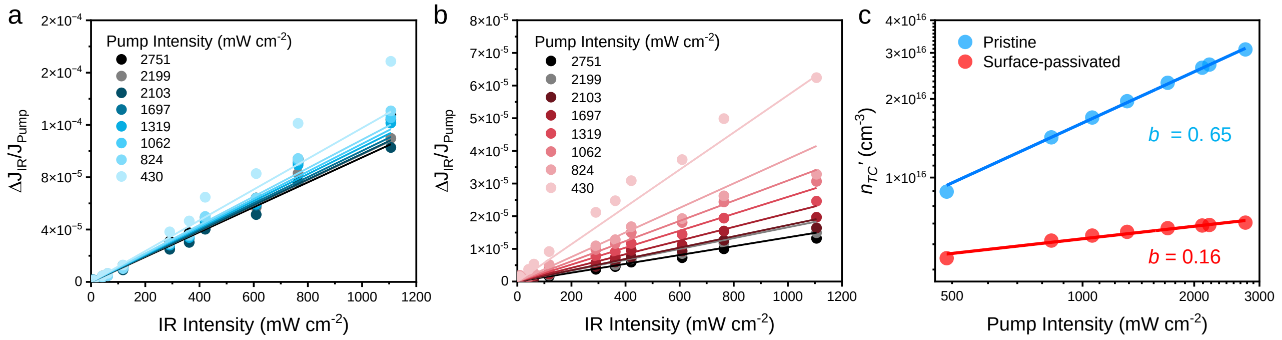

Supplementary Figure 8 | PPPc data analysis based on the kinetic model shown in Supplementary Figure 7 and Supplementary Note 4. (a) Global fitting of $\Delta J_{IR}/J_{Pump}$ data for the pristine device. (b) Global fitting of $\Delta J_{IR}/J_{Pump}$ data for the surface-passivated device. (c) Modelled trapped carrier concentration (n'_{TC}) as a function of pump intensity. The calculated b values (in Equation 1) of pristine and surface-passivated devices are 0.65 and 0.16, respectively.

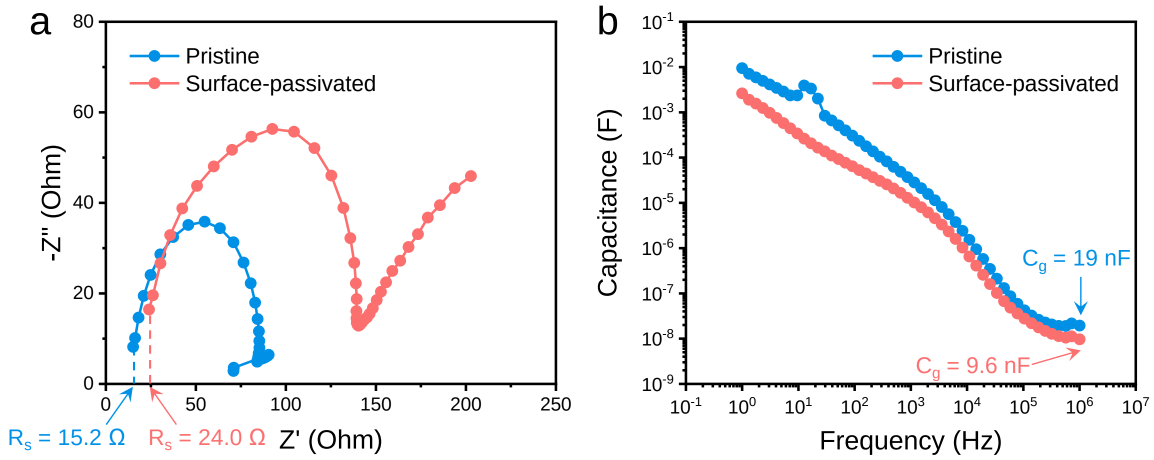

Supplementary Figure 9 | RC constant extraction from pristine and surface-passivated PeSCs measured by electrochemical impedance spectroscopy under white light (100 mW cm^{-2}) at open-circuit voltage condition. (a) Nyquist plots and (b) capacitance as a function of frequency. The series resistance (R_s) of the devices was extracted from the Nyquist plots **a** while the geometric capacitance (C_g) was extracted at 1 MHz from **b**. The calculated RC time constants are 0.29 μs and 0.23 μs for pristine and surface-passivated PeSCs, respectively.

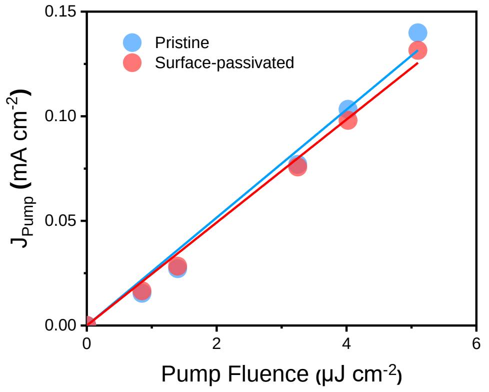

Supplementary Figure 10 | J_{Pump} versus pump fluence curves in ns-PPPc measurement for pristine and surface-passivated devices. Both curves show linear relationships, which suggests first-order (or pseudo-first order) recombination was dominant in the devices at short circuit conditions.

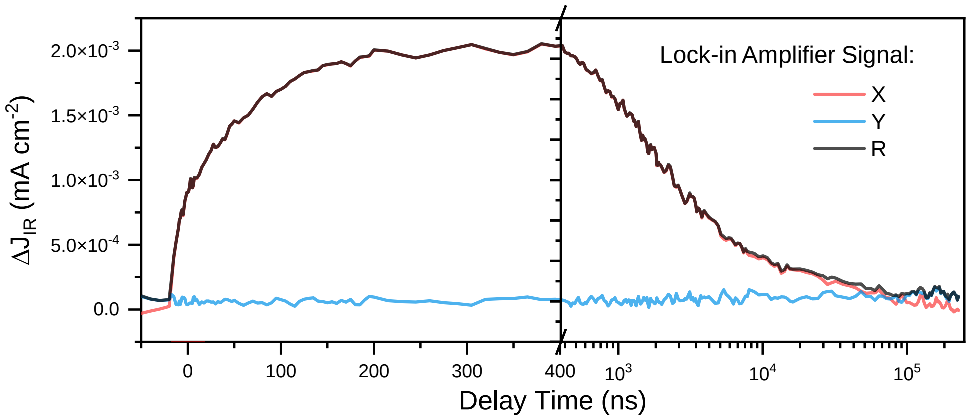

Supplementary Figure 11 | ΔJ_{IR} response of $FA_{0.99}Cs_{0.01}PbI_3$ device under 800-nm pump (5.1 μJ cm^{-2}) and 1064-nm push (170.8 μJ cm^{-2}) in ns-PPPc measurement. The observed 90-degree phase change in time-resolved PPPc measurement after 1×10^5 ns is expected and attributed to the comparable time scale of the electrical delay and the modulation period of the chopper. This effect is accounted for and does not affect the results, when the total amplitude *R* value is presented.

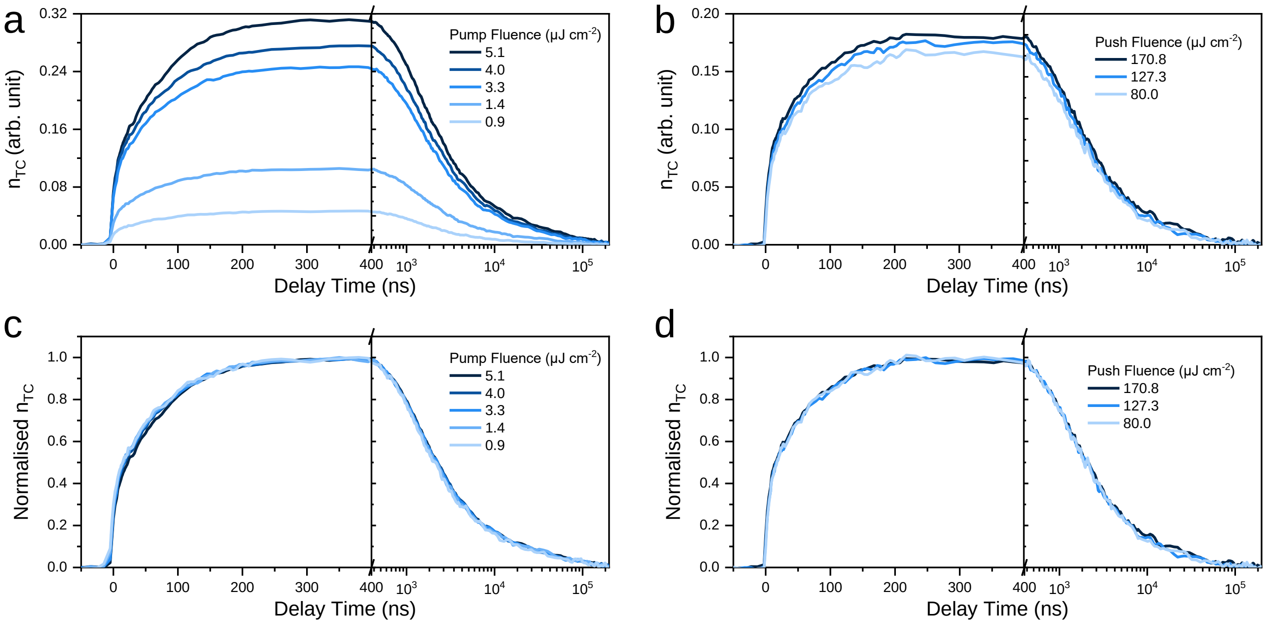

Supplementary Figure 12 | Fluence-dependent n_{TC} dynamics of a pristine device. (a) Pump fluence-dependent results. (b) Push fluence-dependent results. (c) Normalised pump fluence-dependent results. (d) Normalised push fluence-dependent results.

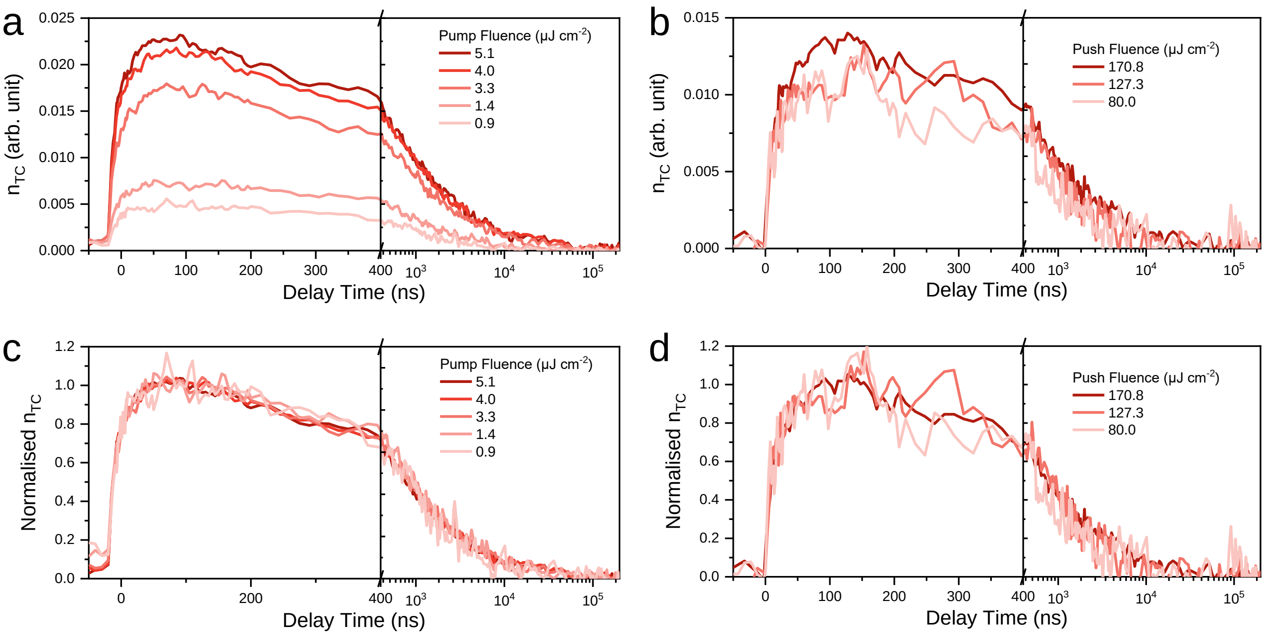

Supplementary Figure 13 | Fluence-dependent n_{TC} dynamics of a surface-passivated device. (a) Pump fluence-dependent results. (b) Push fluence-dependent results. (c) Normalised pump fluence-dependent results. (d) Normalised push fluence-dependent results.

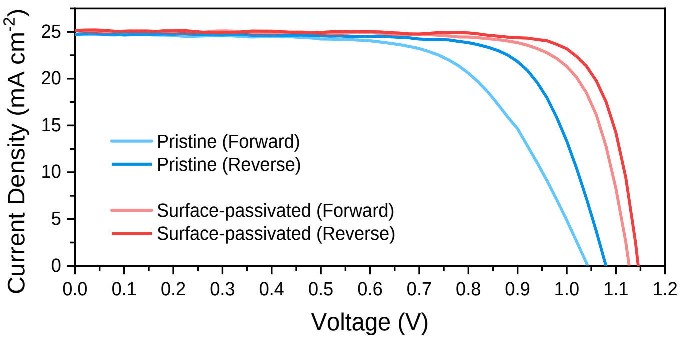

Supplementary Figure 14 | Current density versus voltage curves of optimised pristine and surface-passivated devices with PMMA. Device architecture: ITO/SnO_2/PMMA/$FA_{0.99}Cs_{0.01}PbI_3$/Spiro-OMeTAD/Au (pristine) and ITO/SnO_2/PMMA/$FA_{0.99}Cs_{0.01}PbI_3$/OAI/Spiro-OMeTAD/Au (surface-passivated). Detailed photovoltaic parameters are listed in Supplementary Table 3.

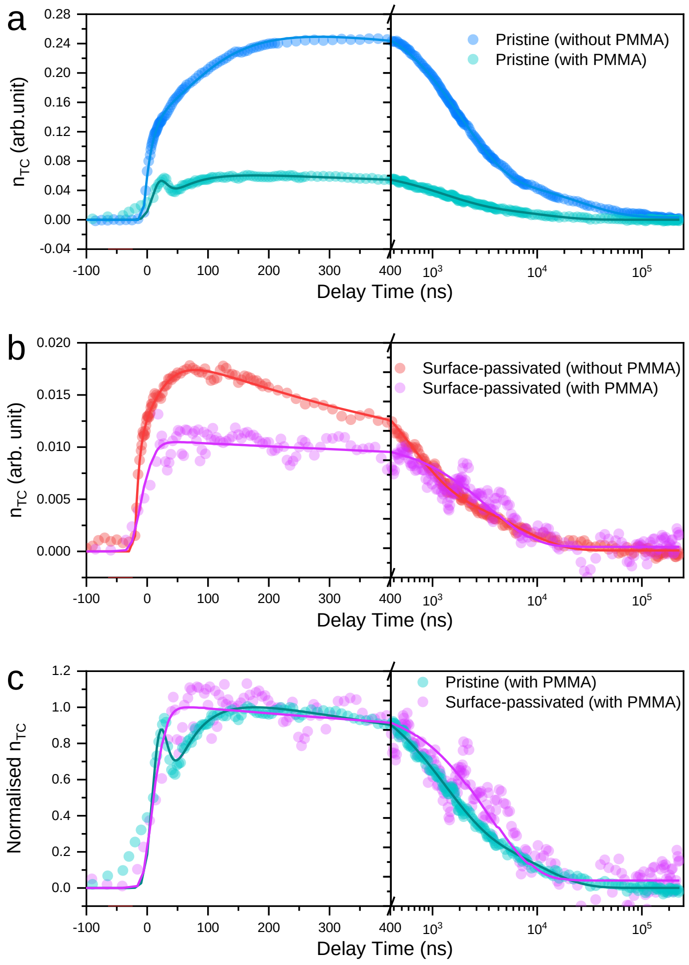

Supplementary Figure 15 | Trapped carrier dynamics of optimised pristine and surface-passivated devices [ITO/SnO_2/PMMA/$FA_{0.99}Cs_{0.01}PbI_3$/(OAI)/Spiro-OMeTAD/Au], under

pump fluence of 3.3 μJ cm^{-2} and push fluence of 170.8 μJ cm^{-2}. (a) Pristine and (b) surface-passivated devices with and without PMMA layer. (c) Normalised dynamics of trapped carriers in pristine and surface-passivated devices with PMMA layer. Note that the spike shown in the pristine device is due to the instant optical effect between pump and push pulses, rather than the occurrence of real trapped carrier dynamics.

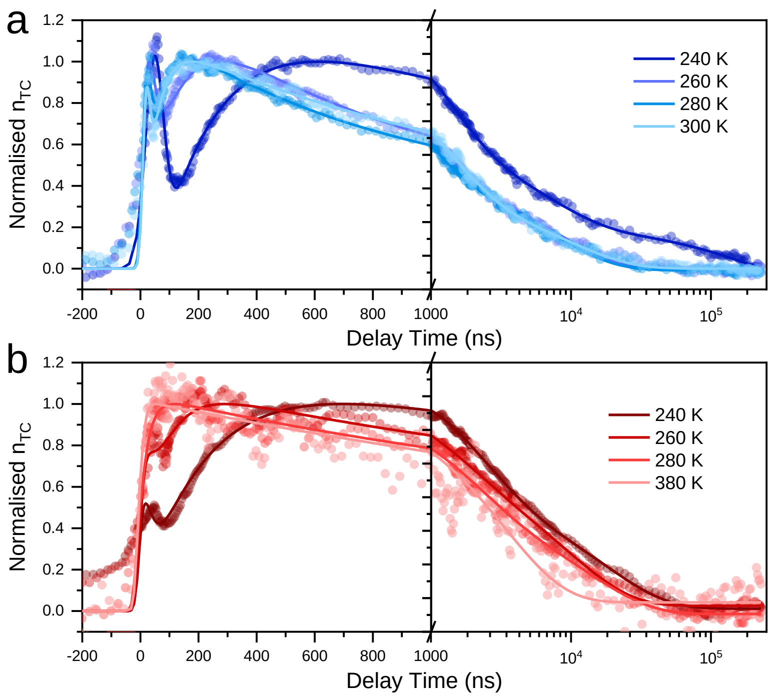

Supplementary Figure 16 | Temperature-dependent results based on different devices. Trapped carrier dynamics of optimised (a) pristine device and (b) surface-passivated device, under pump fluence of 3.3 μJ cm^{-2} and push fluence of 170.8 μJ cm^{-2}. Note that the spikes shown in both cases are due to the instant optical effect between pump and push pulses, rather than the occurrence of real trapped carrier dynamics.

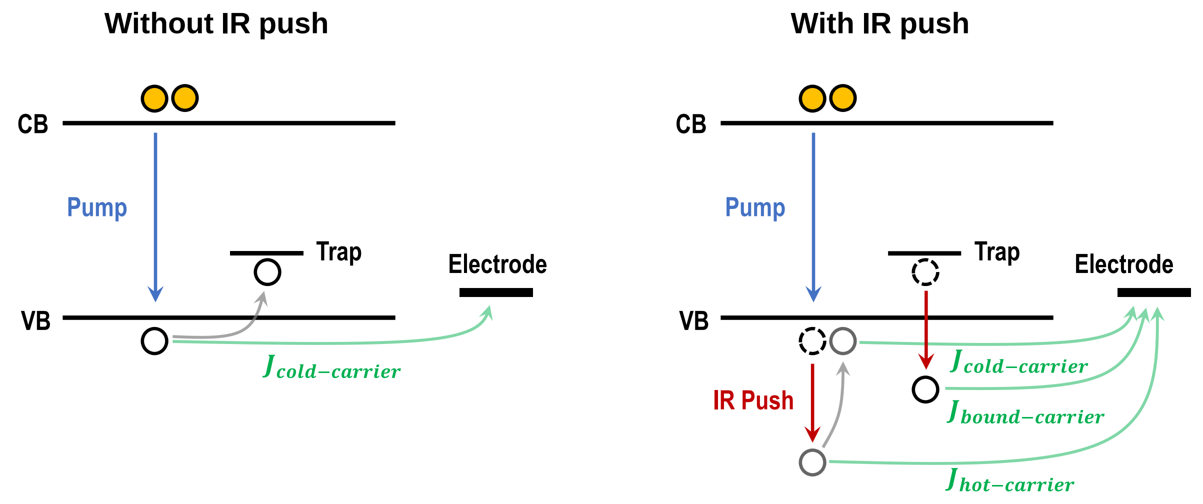

Supplementary Figure 17 | Schematic diagram showing the hole extraction (for current generation) without and with IR push. All the arrows indicate physical processes in terms of holes.

Supplementary Tables

Supplementary Table 1 | Photovoltaic parameters of pristine and surface-passivated $FA_{0.99}Cs_{0.01}PbI_3$ devices.

		V_{oc} (V)	J_{sc} (mA cm^{-2})	FF (%)	PCE (%)
Pristine	Forward	0.99	24.2	53.5	12.8
	Reverse	1.01	24.3	65.4	16.1
Surface-passivated	Forward	1.08	24.3	64.3	16.9
	Reverse	1.09	24.4	73.3	19.5

Supplementary Table 2 | Summary of fitted and fixed parameters in the kinetic model (in Supplementary Note 4) for CW-PPPc. The k_{tn} and k_{tp} are estimated based on the ns-PPPc results (Figure 4a) by assuming an absorption cross-section of 1×10^{17} cm^2 for trapped carriers.

	Fitted Parameters		Fixed Parameters	
Pristine Device	n_{t0} (cm^{-3})	3.85×10^{17}	k_{tn} (cm^3 s^{-1})	1.51×10^{-11}
	k_{btb} (cm^3 s^{-1})	4.53×10^{-9}	k_{tp} (cm^3 s^{-1})	2.42×10^{-10}
	k_E (s^{-1})	9.38×10^5	N_{CB}, N_{VB} (cm^{-3})	1×10^{20}
	R_{Pump} (cm^{-1})	35	T (K)	300
	R_{IR} (cm^2)	5.02×10^{-17}		
Surface-Passivated Device	n_{t0} (cm^{-3})	8.05×10^{15}	k_{tn} (cm^3 s^{-1})	7.10×10^{-11}
	k_{btb} (cm^3 s^{-1})	9.13×10^{-10}	k_{tp} (cm^3 s^{-1})	1.78×10^{-9}
	k_E (s^{-1})	8.26×10^6	N_{CB}, N_{VB} (cm^{-3})	1×10^{20}
	R_{Pump} (cm^{-1})	22450	T (K)	300
	R_{IR} (cm^2)	4.71×10^{-15}		

Supplementary Table 3 | Photovoltaic parameters of optimised pristine and surface-passivated devices.

		V_{oc} (V)	J_{sc} (mA cm^{-2})	FF (%)	PCE (%)
Pristine	Forward	1.04	24.7	64.9	16.7
	Reverse	1.08	24.7	74.3	19.8
Surface-passivated	Forward	1.14	25.1	76.8	21.9
	Reverse	1.15	25.2	80.4	23.2

Supplementary Table 4 | The parameters in drift-diffusion model used for ns-TAS simulations (Supplementary Note 6), that are different from those used in the original Driftfusion code.

	Pristine	Surface-Passivated
B (cm s^{-1})	1×10^{-12}	1×10^{-12}
sn_l (cm s^{-1})	1×10^{-2}	1×10^{-2}
sn_r (cm s^{-1})	1×10^{-3}	1×10^{-3}
sp_l (cm s^{-1})	1×10^{-3}	1×10^{-3}
sp_r (cm s^{-1})	1×10^{-2}	1×10^{-2}
μ (cm^2 V^{-1} s^{-1})	1.8	1.8

Supplementary Table 5 | The parameters in drift-diffusion model used for ns-PPPc simulations (Supplementary Note 6), that are different from those used in the original Driftfusion code.

	Pristine	Surface-Passivated
B (cm s^{-1})	1×10^{-12}	1×10^{-12}
sn_l (cm s^{-1})	1.0	2.8
sn_r (cm s^{-1})	1×10^{-3}	1×10^{-3}
sp_l (cm s^{-1})	1×10^{-3}	1×10^{-3}
sp_r (cm s^{-1})	1.0	2.8
μ (cm^2 V^{-1} s^{-1})	1.8	1.8

Supplementary Table 6 | Summary of parameters used in Equation S6.6 in Supplementary Note 6.

	Pristine	Surface-Passivated
n_{t0} (cm^{-3})	3.85×10^{17}	8.05×10^{15}
k_{tn} (cm^3 s^{-1})	1.5×10^{-13}	6.8×10^{-13}
k_{tp} (cm^3 s^{-1})	2.4×10^{-12}	1.7×10^{-11}
N_{CB}, N_{VB} (cm^{-3})	1×10^{20}	1×10^{20}
T (K)	300	300

Supplementary Note

Supplementary Note 1: Estimation of the impact of IR heat on photocurrent difference.

To estimate how the IR push could affect the temperature of perovskite in CW-PPPc experiments, we estimate its temperature increase (ΔT) resulting from the absorption of IR beam by the following equation:

$$\Delta T = \frac{Q}{m \cdot c} = \frac{A \cdot P_{IR} \cdot \frac{1}{2f_{IR}}}{\rho \cdot V \cdot c} = \frac{A \cdot P_{IR}}{\rho \cdot d \cdot S \cdot c \cdot 2f_{IR}} \qquad (S1.1)$$

Here:

Q is the absorbed energy of IR beam when the chopper is on;

m is the mass of the perovskite;

c is the specific heat capacity of perovskite (308 J kg^{-1} K^{-1}); [1]

A is the percentage of the light absorbed by photogenerated carriers in the active layer of the device (measured using TAS to be below 0.15%);

P_{IR} is the maximum power of 980-nm IR beam in our experiments (i.e., 1.3 mW);

ρ is the density of perovskite (4.1×10^{-3} kg cm^{-3}); [2]

V is the volume of perovskite under IR illumination, which is determined by the product of d (the distance of light passing through the perovskite layer, 1600 nm) and S (beam size of IR light, 0.0012 cm^2);

f_{IR} is the chopper frequency (717 Hz).

In this case, even assuming no heat flows out of the active layer during the illumination, the maximum IR power used increases the temperature of perovskite by 0.0056 K. According to Figure 2c, such a temperature increase could cause a decrease in photocurrent density by 5.5×10^{-6} mA cm^{-2}, which is over 2 orders smaller than the typical PPPc signal (~1×10^{-3} mA cm^{-2}) at the corresponding pump and push intensity (Supplementary Figure 4). Note that this estimation does not consider contributions from substrate heating by the IR beam, because the transfer of heat from the substrate to the perovskite would have a time lag which would manifest itself as a decreased out-of-phase (Y) component of the signal (according to Figure 2c) at lower frequencies which, however, was not observed in Supplementary Figure 3. Hence, we consider that heat generated by the IR push has a negligible effect on the PPPc results in our study.

Supplementary Note 2: Origin of PPPc signal

Supplementary Figure 17 illustrates the fundamental operating principle of PPPc technique. Under optical illumination a photocurrent density $J_{without-IR}$ is extracted from the device. This photocurrent is composed from thermalised (cold) free carriers so that $J_{without-IR} = J_{cold-carrier}$. When the IR push beam illuminates the device and a new photocurrent density $J_{with-IR}$ is observed. Some trapped carriers are optically detrapped by the IR and contribute an additional component $J_{bound-carrier}$to the photocurrent density. Band-edge free carriers will also be excited to higher energy states and some these will also contribute an amount $J_{hot-carrier}$ to $J_{with-IR}$. However, this reduces the population of thermalised carriers contributing to $J_{with-IR}$ by a similar amount so that $J_{cold-carrier} = J_{without-IR} - J_{hot-carrier}$. Since most hot carriers quickly cool back to band-edge to become cold carriers typically within 1 ps,[3] much faster than the time scale for charge collection at the contacts (~100 ns, see Figure 4), so we would expect $J_{hot-carrier}$ to be very small. Our previous research has also shown that hot carrier trapping generally is not present in 3D perovskites, so we would not expect its occurrence here.[4,5] Therefore, the additional photocurrent density induced by the IR beam is given by:

$$\begin{aligned}\Delta J_{IR} &= J_{with-IR} - J_{without-IR} \\ &= [J_{hot-carrier} + (J_{without-IR} - J_{hot-carrier}) + J_{bound-carrier}] - J_{without-IR} \\ &= J_{bound-carrier} \qquad \text{(S2.1)}\end{aligned}$$

The high selectivity of PPPc, which is only sensitive to bound species in the device and make unbound species undetectable, has made it a valuable tool in the investigation of organic solar cells over the past decade.[6] In organic solar cells, bound species typically refer to excitons and traps, while in 3D perovskite solar cells, the bound species are solely traps since negligible excitons are present due to their small exciton binding energy.

Supplementary Note 3: Calculation of the trapped carrier concentration (n_{TC}) from intensity-dependent CW-PPPc results.

To better understand the amplitude of ΔJ_{IR} signal, we can evaluate it by the following expression:

$$\Delta J_{IR} = \frac{I_{IR} \cdot \lambda_{IR}}{hc} \cdot (1 - e^{-\alpha_{IR} \cdot d}) \cdot E(I_{Pump}) \cdot q \quad \text{(S3.1)}$$

where I_{IR} is the push intensity before chopper; λ_{IR} is the push wavelength; α_{IR} is the absorption coefficient of trapped carriers which depends on the number of trapped carriers present; q is the elementary charge; h is Planck's constant; c is the speed of light; $E(I_{Pump})$ is the charge extraction possibility, which is a function of pump intensity (I_{Pump}) due to the change of dominant recombination process (i.e., first-order trap-mediated in bulk or at interface, band-to-band, or Auger recombination) and charge distribution resulting from different concentrations of band-edge carriers. Note that the increased concentration of band-edge carriers generated by the IR push beam is small compared to the population of band-edge carriers generated by the pump beam. Therefore, the charge extraction possibility in the "pump only" and "pump + push" cases is assumed to be identical.

We can also evaluate J_{Pump} by the following expression:

$$J_{Pump} = \frac{I_{Pump} \cdot \lambda_{Pump}}{hc} \cdot (1 - e^{-\alpha_{Pump} \cdot d}) \cdot E(I_{Pump}) \cdot q \quad \text{(S3.2)}$$

where λ_{Pump} and α_{Pump} are pump wavelength and corresponding absorption coefficient, respectively. Note that the intrinsic absorption coefficients of the 800/808-nm pump and 980-nm push beams for $FAPbI_3$, which are reported from literatures, are ~3×10^3 cm^{-1} (α_{Pump}) and << 1 m^{-1}, respectively.[7,8] Based on these values, we can infer that the pump has a penetration depth of ~3.33 μm, while the IR push has a much larger penetration depth of >> 1 m (in the absence of free carriers), suggesting that both of them can reach the top surface of perovskite.

Combining Equations S3.1 and S3.2, we can calculate $\Delta J_{IR}/J_{Pump}$ to eliminate the unknown parameter of $E(I_{Pump})$:

$$\frac{\Delta J_{IR}}{J_{Pump}} = \frac{\frac{I_{IR} \cdot \lambda_{IR}}{hc} \cdot (1 - e^{-\alpha_{IR} \cdot d}) \cdot E(I_{Pump}) \cdot q}{\frac{I_{Pump} \cdot \lambda_{Pump}}{hc} \cdot (1 - e^{-\alpha_{Pump} \cdot d}) \cdot E(I_{Pump}) \cdot q} = \frac{(1 - e^{-\alpha_{IR} \cdot d}) \cdot I_{IR} \cdot \lambda_{IR}}{(1 - e^{-\alpha_{Pump} \cdot d}) \cdot I_{Pump} \cdot \lambda_{Pump}}$$

$$= \frac{(1 - e^{-\sigma \cdot n_{TC} \cdot d}) \cdot \lambda_{IR}}{(1 - e^{-\alpha_{Pump} \cdot d}) \cdot I_{Pump} \cdot \lambda_{Pump}} \cdot I_{IR} \quad \text{(S3.3)}$$

where σ is the absorption cross-section of trapped carriers and $\alpha_{IR} = \sigma \cdot n_{TC}$, while n_{TC} refers to the trapped carrier concentration. Here, σ originates from the intrinsic properties of trapped carriers, which should solely relate to the types of traps.[9] In such a case, we consider σ, at a specific push wavelength, to be a constant. And it is identical for the 800-nm thick pristine and surface-passivated $FA_{0.99}Cs_{0.01}PbI_3$, because OAI passivation only changed the density rather than the type of traps (as

the depth of traps is similar, as discussed in the main text). Therefore, we can simplify the discussion via the relationship of $\alpha_{IR} \propto n_{TC}$. Finally, n_{TC} was assumed to be homogenous across the whole perovskite film, and hence demonstrates the average effect from both bulk and surface traps of perovskite.

Based on Equation S3.3, we see that $\Delta J_{IR}/J_{Pump}$ depends linearly on I_{IR} if the fractional changes in n_{TC} induced by I_{IR} are small. This assumption is consistent with the results shown in Figures 3a and S5. In such a case, the slope of $(\Delta J_{IR}/J_{Pump})$ *vs* I_{IR} can be expressed by the following equation, which can also be quantitatively extracted from Figures 3a and S5.

$$\frac{d\left(\Delta J_{IR}/J_{Pump}\right)}{dI_{IR}} = \frac{\left(1 - e^{-\alpha_{IR}\cdot d}\right)\cdot \lambda_{IR}}{\left(1 - e^{-\alpha_{Pump}\cdot d}\right)\cdot I_{Pump}\cdot \lambda_{Pump}} \tag{S3.4}$$

Therefore, n_{TC} can be expressed as a function of I_{Pump}:

$$n_{TC} \propto \alpha_{IR} = -\frac{1}{d}\cdot ln\,\{1 - \frac{\left(1 - e^{-\alpha_{Pump}\cdot d}\right)\cdot I_{Pump}\cdot \lambda_{Pump}}{\lambda_{IR}}\cdot \frac{d\left(\Delta J_{IR}/J_{Pump}\right)}{dI_{IR}}\} \tag{S3.5}$$

where the slope term can be determined from the relevant $(\Delta J_{IR}/J_{Pump})$ *vs* I_{IR} dependence measured experimentally.

Supplementary Note 4: Kinetic Model for CW-PPPc.

As discussed in the main text, we considered hole traps dominated the total trap states of perovskite. In such a scenario, to simplify the kinetic model, a single trap level is considered here. Supplementary Figure 7 shows a zero-dimensional kinetic model for CW-PPPc which can be described by the following equations:

$$\frac{dn}{dt} = \frac{R_{Pump}\cdot \lambda_{Pump}}{hc} I_{Pump} - k_E n - k_{btb}(np - n_i^2) - k_{tn}\left[nn'_{TC} - N_{CB}e^{\frac{q(E_t - E_{CB})}{k_B T}}(n_{t0} - n'_{TC})\right] \tag{S4.1}$$

$$\frac{dn'_{TC}}{dt} = k_{tp}\left[p(n_{t0} - n'_{TC}) - N_{VB}e^{\frac{q(E_{VB} - E_t)}{k_B T}} n'_{TC}\right] - k_{tn}\left[nn'_{TC} - N_{CB}e^{\frac{q(E_t - E_{CB})}{k_B T}}(n_{t0} - n'_{TC})\right] - \frac{R_{IR}\cdot \lambda_{IR}}{hc} n'_{TC} I_{IR}(t) \tag{S4.2}$$

$$\frac{dp}{dt} = \frac{R_{Pump} \cdot \lambda_{Pump}}{hc} I_{Pump} - k_E p - k_{btb}(np - n_i^2) - k_{tp}\left[p(n_{t0} - n'_{TC}) - N_{VB} e^{\frac{q(E_{VB}-E_t)}{k_B T}} n'_{TC}\right] + \frac{R_{IR} \cdot \lambda_{IR}}{hc} n'_{TC} I_{IR}(t) \quad \text{(S4.3)}$$

where, in this context, n, p, and n'_{TC} represent the average concentration of electrons, holes, and trapped holes, respectively, and $n_i = \sqrt{N_{CB}N_{VB}}\, exp[q(E_{VB} - E_{CB})/(2k_B T)]$ is the intrinsic carrier concentration. These quantities are averaged for the thickness of the film regardless of whether the charges are located at interfaces or in the bulk. I_{Pump} and $I_{IR}(t)$ are the intensity of pump (constant) and push beam (square wave) respectively, the push beam is assumed to only excite holes from the traps; R_{Pump} and R_{IR} are the absorption factors for the pump and push beams respectively; n_{t0} is the trap density; k_{btb} is the band-to-band recombination rate constant. k_{tn} and k_{tp} are the electron and hole capture rate constants. k_E is the extraction rate constant for carrier (for simplicity, assumed to be the same for electrons and holes) to the electrodes. $E_t - E_{CB}$ (−1.46 eV) and $E_{VB} - E_t$ (−0.28 eV) are the trap energy relative to the conduction and valence band, determined from the trap activation energy measurements (Figure 3d) and the band gap. k_B and T are Boltzmann constant and temperature, respectively. And Euler's method is used to resolve the dynamics as a function of time.

To investigate the relationship between modelled trapped carrier concentration and pump intensity (n'_{TC} versus I_{Pump}), we globally fitted the $\Delta J_{IR}/J_{Pump}$ results in Figures 3a and S5. The detailed parameters setting for the global fitting and fitted parameters are listed in Supplementary Table 2 and the fitted results are shown in Figures S8a and S8b. Putting all parameters back into the kinetic model allows us to model the n'_{TC} versus I_{Pump} curves for pristine and surface-passivated devices (Supplementary Figure 8c). While the fitted n_{t0} allows us to estimate the trap density ratio between pristine and surface-passivated devices.

Supplementary Note 5: Calculation of the trapped carrier concentration from ns-PPPc results.

Similar to the analysis for CW-PPPc results, the ΔJ_{IR} signal as a function of time (t) [i.e., $\Delta J_{IR}(t)$] can be expressed as:

$$\Delta J_{IR}(t) = \frac{\frac{I_{IR}}{f_{IR}} \cdot \lambda_{IR}}{hc} \cdot \left[1 - e^{-\alpha_{IR}(t)\cdot d}\right] \cdot E\left(I_{Pump}, t\right) \cdot q \quad \text{(S5.1)}$$

In this context, f_{IR} is the repetition rate of the IR laser and I_{IR} refers to the time averaged intensity of the train of push pulses. In addition, note that the J_{Pump} has a linear relationship with I_{Pump}

(Supplementary Figure 10), which suggests that first-order recombination dominates the total recombination in the measuring range. In this a case, since $\Delta J_{IR}/J_{Pump}$ is small, the charge extraction probability $E(I_{Pump}, t)$ is a constant (E) and not dependent on I_{Pump} and t. Therefore, $\Delta J_{IR}(t)$ and J_{Pump} measured by the lock-in amplifier can be expressed as:

$$\Delta J_{IR}(t) = \frac{\frac{I_{IR}}{f_{IR}} \cdot \lambda_{IR}}{hc} \cdot \left[1 - e^{-\alpha_{IR}(t)\cdot d}\right] \cdot E \cdot q \tag{S5.2}$$

$$J_{Pump} = \frac{\frac{I_{Pump}}{f_{pump}} \cdot \lambda_{Pump}}{hc} \cdot \left(1 - e^{-\alpha_{Pump}\cdot d}\right) \cdot E \cdot q \tag{S5.3}$$

where f_{pump} is the repetition rate of the pump pulses, which is identical to f_{IR} (i.e., 4000 Hz), and I_{pump} refers to the time averaged intensity of the pump pulses. We can combine Equations S5.2 and S5.3 to calculate $\Delta J_{IR}/J_{Pump}$ to eliminate the E:

$$\frac{\Delta J_{IR}}{J_{Pump}}(t) = \frac{\frac{I_{IR} \cdot \lambda_{IR}}{f_{IR} \cdot hc} \cdot \left[1 - e^{-\alpha_{IR}(t)\cdot d}\right] \cdot E \cdot q}{\frac{I_{pump} \cdot \lambda_{Pump}}{f_{pump} \cdot hc} \cdot \left(1 - e^{-\alpha_{Pump}\cdot d}\right) \cdot E \cdot q} = \frac{I_{IR} \cdot \lambda_{IR} \cdot \left[1 - e^{-\alpha_{IR}(t)\cdot d}\right]}{I_{Pump} \cdot \lambda_{Pump} \cdot \left(1 - e^{-\alpha_{Pump}\cdot d}\right)} \tag{S5.4}$$

Therefore,

$$n_{TC}(t) \propto \alpha_{IR}(t) = -\frac{1}{d} \cdot ln\left[1 - \frac{I_{Pump} \cdot \lambda_{Pump} \cdot \left(1 - e^{-\alpha_{Pump}\cdot d}\right)}{I_{IR} \cdot \lambda_{IR}} \cdot \frac{\Delta J_{IR}}{J_{Pump}}(t)\right] \tag{S5.5}$$

Supplementary Note 6: Simulated the concentration of free holes and trapped holes for ns-TAS and ns-PPPc spectroscopies.

Driftfusion is an open-source MATLAB based simulation package describing semiconducting devices in one dimension. The software solves a set of semi-classic partial differential equations using MATLABs (PDEPE solver).[10,11] The code used for the simulation is available in: https://github.com/barnesgroupICL/Driftfusion. The model simulates semiconductor devices using a coupled set of continuity equations to determine solutions for the time- and position-dependent electrostatic potential $[V(x,t)]$ described by Poisson equation and the free electron $n(x,t)$ and free hole $p(x,t)$ densities. Also, ionic species (optional) are denoted as $c(x,t)$ for cation and $a(x,t)$ for anion. The equations account for the drift due to the electric field in the layer (gradient of electrical potential) and the diffusion due to gradients in electron and hole concentration. Recombination and mobile ionic defects are also considered. All equations are given as follows:

$$C \cdot \frac{\partial u}{\partial t} = x^{-m} \frac{\partial}{\partial x}(x^m f) + s \quad \text{(S6.1)}$$

In which:

$$u = \begin{bmatrix} V \\ n \\ p \\ c \\ a \end{bmatrix} \; C = \begin{bmatrix} 0 \\ 1 \\ 1 \\ 1 \\ 1 \end{bmatrix} \; f = \begin{bmatrix} \frac{\epsilon_r(x)}{\epsilon_{max}} \frac{dV}{dx} \\ -j_n \\ -j_p \\ -j_c \\ -j_a \end{bmatrix} \; s = \begin{bmatrix} \frac{q}{\epsilon_{max}\epsilon_0}(p(x,t) - n(x,t) + c(x,t) - a(x,t) + N_i(x)) \\ g_n(x,t) - r_n\,(x,t) \\ g_p(x,t) - r_p(x,t) \\ 0 \\ 0 \end{bmatrix} \quad \text{(S6.2)}$$

where $\epsilon_r(x)$ is relative permittivity normalised by ϵ_{max}, q is the unit charge, g_n, g_p are the carrier generation rate under laser excitation (the spatial dependence origin from beer-lambert law). j_n, j_p, j_c, j_a denote currents, and r_n, r_p denote recombination rates for electrons, holes, cations, and anions, respectively. $N_i(x)$ are static ionic densities including $N_A, N_D, N_{cat}, N_{ani}$.

Equations S6.3–6.5 show the generalised form for boundary conditions. For $V(x,t)$, Dirichlet boundary condition is applied since the electrode of device has fixed potential after connection. V_r, V_l, V_{app}, and V_{bi} are the potential of right electrode, the potential of left electrode, the applied bias, and the built-in potential difference, respectively. n_{0l}/p_{0l} denotes the initial electron/hole concentration on left boundary and so as n_{0r}/p_{0r} for right boundary. For $c(x,t)$ and $a(x,t)$, the boundary is zero flux. For $n(x,t)/p(x,t)$, Neumann boundary conditions are applied through a fixed extraction rate of electron ($sn_{l,r}$) and a fixed extraction rate of hole ($sp_{l,r}$):

$$p(x,t,u) + q(x,t) f\left(x,t,u,\frac{du}{dx}\right) = 0 \quad \text{(S6.3)}$$

In which:

$$u = \begin{bmatrix} V \\ n \\ p \\ c \\ a \end{bmatrix} \quad p_l = \begin{bmatrix} -V_l \\ -sn_l\left(n(0) - n_{0_l}\right) \\ -sp_l\left(p(0) - p_{0_l}\right) \\ 0 \\ 0 \end{bmatrix} \; ql = \begin{bmatrix} 0 \\ 1 \\ 1 \\ 1 \\ 1 \end{bmatrix} \quad \text{(S6.4)}$$

$$p_r = \begin{bmatrix} -V_r + V_{bi} - V_{app} - V_{res} \\ -sn_r\left(n(x_{max}) - n_{0_r}\right) \\ -sp_l\left(p(x_{max}) - p_{0_r}\right) \\ 0 \\ 0 \end{bmatrix} \; qr = \begin{bmatrix} 0 \\ 1 \\ 1 \\ 1 \\ 1 \end{bmatrix} \quad \text{(S6.5)}$$

The sample is modelled with a single active layer and the electron and hole transport layers are simplified by extraction rate and fermi-level difference on both sides. Fermi-level, ionisation potential, and electron affinity etc. are all taken from preset values given in Ref. [10]. And the changed parameters are detailed in follows section.

For ns-TAS simulation: To match the time scale of the simulated TAS result and experimental TAS data. We adjusted the values of carrier mobility, extraction, and recombination rate B used in Driftfusion (Equation S6.2) to fit the simulated average of the electron $[n(x,t)]$ and hole $[p(x,t)]$ concentration profiles (normalised and plotted against time), with those inferred from the ns-TAS measurements from the ground state bleach decay (Figure 4b). The recombination rate B relates to band-to-band recombination, and it is the main recombination we considered in the bulk with a relationship of $r(x,t) \propto B[n(x,t)p(x,t)]$.

All parameters different from those used in the original Driftfusion code in Ref. [10] are listed in Supplementary Table 4.

For ns-PPPc result simulation: To explicitly estimate the dynamics of trapped carriers at the interface, we calculated solutions for the conduction and valence band carriers in the perovskite layer as a function of time and position following an optical pump laser pulse as described above. We then used these solutions as a basis for estimating the variation in time of the number of trapped carriers at the interface. This approach is reasonable since the total concentration of trapped carriers is several orders of magnitude smaller than the concentrations of free carriers in the conduction and valence bands which will not be significantly perturbed. The trapping dynamics for PPPc are realised by using the surface concentration of free carriers as a function of time as an input to a simplified ordinary differential equation (ODE) that forms the basis of the Shockley–Read–Hall rate equation to solve for the time dependent concentration of trapped charge.[10,11] Instead of using a Shockley–Read–Hall recombination rate as implemented in the original drift-diffusion code, we directly utilised a separate ODEs to solve for the time-dependent concentration of trapped holes n'_{TC} at the HTL interface.

$$\frac{dn'_{TC}}{dt} = k_{tp}p_l(n_{t0} - n'_{TC}) - k_{tp}N_{VB}e^{\frac{q(E_{VB}-E_t)}{k_BT}}n'_{TC} - k_{tn}n_l n'_{TC} + k_{tn}N_{CB}e^{\frac{q(E_t-E_{CB})}{k_BT}}(n_{t0} - n'_{TC}) \quad (S6.6)$$

where $n_l(t)$ and $p_l(t)$ are the concentrations of free electrons and holes at the HTL interface (at x = 0, left), respectively, determined from the Driftfusion solution following a simulated laser pulse. n_{t0} is the concentration of trap states, k_{tn} and k_{tp} are the electron and hole capture rate constants, N_{VB} and N_{CB} are the effective density of states at the valence and conduction band edges and $E_{VB} - E_t$ (−0.28 eV) and $E_t - E_{CB}$ (−1.46 eV) are the trap depth relative to the valence band edge and conduction band edges, respectively, determined from the trap activation energy measurements (Figure 3d) and the band gap.

Then we performed Driftfusion simulation by adding carrier extraction to both electrodes. We selected the solution data for electron and hole concentration at the HTL interface, $n_l(t)$ and $p_l(t)$, and applied it to our ODE (Equation S6.6) to simulate the ns-PPPc measurement. This approach is

based on the assumption that the PPPc signal is significantly associated with trapped holes at the interface. The change of photocurrent density $\Delta J_{IR}/J_{Pump}$ directly relates to the transient free electron and hole concentrations at the interface.

All the parameters different from those used in the original Driftfusion code in Ref. [10] are listed in Supplementary Table 5. And the parameters used for Equation S6.6 are summarised in Supplementary Table 6.

Supplementary References

1. Du, X. *et al.* Lead halide perovskite for efficient optoacoustic conversion and application toward high-resolution ultrasound imaging. *Nat. Commun.* **12**, 3348 (2021).

2. Stoumpos, C. C., Malliakas, C. D. & Kanatzidis, M. G. Semiconducting tin and lead iodide perovskites with organic cations: phase transitions, high mobilities, and near-infrared photoluminescent properties. *Inorg. Chem.* **52**, 9019–9038 (2013).

3. Villamil Franco, C. *et al.* Exciton cooling in 2D perovskite nanoplatelets: rationalized carrier-induced stark and phonon bottleneck effects. *J. Phys. Chem. Lett.* **13**, 393–399 (2022).

4. Hopper, T. R. *et al.* Hot carrier dynamics in perovskite nanocrystal solids: role of the cold carriers, nanoconfinement, and the surface. *Nano Lett.* **20**, 2271–2278 (2020).

5. Hopper, T. R. *et al.* Ultrafast intraband spectroscopy of hot-carrier cooling in lead-halide perovskites. *ACS Energy Lett.* **3**, 2199–2205 (2018).

6. Zhang, J. *et al.* Efficient non-fullerene organic solar cells employing sequentially deposited donor–acceptor layers. *J. Mater. Chem. A* **6**, 18225–18233 (2018).

7. Wright, A. D. *et al.* Intrinsic quantum confinement in formamidinium lead triiodide perovskite. *Nat. Mater.* **19**, 1201–1206 (2020).

8. Xie, Z. *et al.* Refractive index and extinction coefficient of $NH_2CH{=}NH_2PbI_3$ perovskite photovoltaic material. *J. Phys.: Condens. Matter* **29**, 245702 (2017).

9. Glunz, S. W. & Preu, R. 1.14 - Crystalline silicon solar cells – state-of-the-art and future developments. *Comprehensive Renewable Energy (Second Edition)* **1**, 293–324 (2022).

10. Calado, P. *et al.* Driftfusion: an open source code for simulating ordered semiconductor devices with mixed ionic-electronic conducting materials in one dimension. *J. Comput. Electron.* **21**, 960–991 (2022).

11. Calado, P. *et al.* Correction to: Driftfusion: an open source code for simulating ordered semiconductor devices with mixed ionic-electronic conducting materials in one dimension. *J. Comput. Electron.* **21**, 1189–1189 (2022).
