# Supplementary material for: Operando dynamics of trapped carriers in perovskite solar cells observed via infrared optical activation spectroscopy
Source: Nat Commun. 2023 Dec 4;14:8000. doi: 10.1038/s41467-023-43852-5 (PMC10694143; doi:10.1038/s41467-023-43852-5)
Supplement: Supplementary file 3 — Reporting Summary [file 41467_2023_43852_MOESM3_ESM.pdf]

nature research

Corresponding author(s): Ziming Chen

Solar Cells Reporting Summary

Nature Research wishes to improve the reproducibility of the work that we publish. This form is intended for publication with all accepted papers reporting the characterization of photovoltaic devices and provides structure for consistency and transparency in reporting. Some list items might not apply to an individual manuscript, but all fields must be completed for clarity.

For further information on Nature Research policies, including our data availability policy, see Authors & Referees.

Experimental design

Please check: are the following details reported in the manuscript?

1. Dimensions

Item	Yes / No	Details
Area of the tested solar cells	☒ Yes ☐ No	Methods: Perovskite film and device characterisation
Method used to determine the device area	☒ Yes ☐ No	Methods: Device fabrication

2. Current-voltage characterization

Item	Yes / No	Details
Current density-voltage (J-V) plots in both forward and backward direction	☒ Yes ☐ No	Figure 1b
Voltage scan conditions *For instance: scan direction, speed, dwell times*	☒ Yes ☐ No	Methods: Perovskite film and device characterisation
Test environment *For instance: characterization temperature, in air or in glove box*	☒ Yes ☐ No	Methods: Perovskite film and device characterisation
Protocol for preconditioning of the device before its characterization	☐ Yes ☒ No	The device was measured without preconditioning
Stability of the J-V characteristic *Verified with time evolution of the maximum power point or with the photocurrent at maximum power point; see ref. 7 for details.*	☐ Yes ☒ No	Our work mainly focuses on the in-depth understanding of trap states in the device, rather than the performance of the device. However, we can provide this information if it is really needed.

3. Hysteresis or any other unusual behaviour

Item	Yes / No	Details
Description of the unusual behaviour observed during the characterization	☐ Yes ☒ No	No unusual behaviour
Related experimental data	☒ Yes ☐ No	We have reported the hysteresis measurement in Figure 1b and Figure S14

4. Efficiency

Item	Yes / No	Details
External quantum efficiency (EQE) or incident photons to current efficiency (IPCE)	☐ Yes ☒ No	Our work mainly focuses on the in-depth understanding of trap states in the device, rather than the performance of the device. However, we can provide this information if it is really needed.
A comparison between the integrated response under the standard reference spectrum and the response measure under the simulator	☐ Yes ☒ No	As we do not have the above EQE data
For tandem solar cells, the bias illumination and bias voltage used for each subcell	☐ Yes ☒ No	Not applicable

5. Calibration

Item	Yes / No	Details
Light source and reference cell or sensor used for the characterization	☒ Yes ☐ No	Methods: Perovskite film and device characterisation
Confirmation that the reference cell was calibrated and certified	☐ Yes ☒ No	The reference cell was already well calibrated when we bought it. We can provide this information if it is really needed.

Calculation of spectral mismatch between the reference cell and the devices under test

- [ ] Yes
- [x] No

Our work mainly focuses on the in-depth understanding of trap states in the device, rather than the performance of the device. However, we can provide this information if it is really needed.

6. Mask/aperture

Size of the mask/aperture used during testing

- [x] Yes
- [ ] No

Methods: Device fabrication

Variation of the measured short-circuit current density with the mask/aperture area

- [ ] Yes
- [x] No

We did not change the mask area

7. Performance certification

Identity of the independent certification laboratory that confirmed the photovoltaic performance

- [ ] Yes
- [x] No

Our work mainly focuses on the in-depth understanding of trap states in the device, rather than the performance of the device. Relative comparison between different devices is accurate enough for our research. Therefore, we consider it is not necessary to report certificated results.

A copy of any certificate(s)
Provide in Supplementary Information

- [ ] Yes
- [x] No

As we do not have certificated results

8. Statistics

Number of solar cells tested

- [ ] Yes
- [x] No

Our work mainly focuses on the in-depth understanding of trap states in the device, rather than the performance of the device. However, we can provide this information if it is really needed.

Statistical analysis of the device performance

- [ ] Yes
- [x] No

As we do not have statistical results

9. Long-term stability analysis

Type of analysis, bias conditions and environmental conditions
For instance: illumination type, temperature, atmosphere humidity, encapsulation method, preconditioning temperature

- [ ] Yes
- [x] No

Our work mainly focuses on the in-depth understanding of trap states in the device, rather than the performance of the device. We consider it is not necessary to report these long-term stability results.
